# Sectionalism

**Published:** 1860-12

**Authors:** 


SECTIONALISM.—The Medical Department of the University of Louisiana having wisely made selection of Prof. Lawson, of Cincinnati, to fill one of its most important chairs, and Prof. Flint, of Buffalo, having previously filled a similar position in that city, the *Oglethorpe Medical and Surgical Journal* is indignant. It says: "The insinuation that "Northern Professors" are even the equal of Southern Professors in teaching 'practical medicine' is wholly gratuitous and not sustained by reliable evidence." In short, "it is very tolerable and not to be endured." Again, it says: "We stand as little in need of Northern medical men at the South, as we do of wooden nutmegs or of Northern politicians." We confess a little surprise at this attempt to make medicine a "local institution." We admit that we shall have to re-read the books before we can be led to believe that science is other than cosmopolitan. Is the attraction of gravitation reversed "down South?"

